# The association between the advanced lung cancer inflammation index and successful antegrade recanalization in patients with coronary chronic total occlusion: a retrospective cross-sectional study

**DOI:** 10.1186/s12872-026-05972-w

**Published:** 2026-05-27

**Authors:** Bingyang Qi, Mengyang Liu, Xin Yi, Yanyao Jia, Yahang Tan, Zhiyong Zhang, Tao Zhang, Zhichang Zheng, Chuang Li, Yuan Fu, Jing Cheng, Lin Zhao

**Affiliations:** 1 Department of Cardiology, Beijing Chaoyang Hostpital, Capital Medical University, Beijing, 100027 China; 2 Department of Endocrinology, Chuiyangliu Hospital Affiliated to Tsinghua University, Beijing, 100027 China; 3 Department of Internal Medicine, Faculty of Medicine and Health Sciences, Universiti Putra Malaysia, Serdang, 43400 Selangor Malaysia

**Keywords:** Chronic total occlusion, Antegrade recanalization, Advanced lung cancer inflammation index, Percutaneous coronary intervention

## Abstract

**Background:**

Chronic total occlusion (CTO) represents one of the most challenging lesion subsets in coronary artery disease, and antegrade recanalization (AR) is the preferred approach for revascularization due to its lower complication risk. However, predicting AR success remains clinically difficult. The Advanced Lung Cancer Inflammation Index (ALI), integrating body mass index, albumin, and neutrophil-to-lymphocyte ratio, reflects nutritional and inflammatory status, both of which may influence procedural outcomes. This study aimed to investigate the association between ALI and successful AR in CTO patients undergoing percutaneous coronary intervention (PCI).

**Methods:**

In this retrospective cross-sectional study, 198 patients with angiographically confirmed CTO undergoing PCI between May 2024 and December 2025 were analyzed. Patients were stratified into successful AR (n = 132) and failed AR (n = 66) groups based on the final recanalization route. ALI was calculated preprocedurally. Multivariable logistic regression, generalized additive modeling (GAM), and subgroup and sensitivity analyses were performed to examine the association between ALI and AR success. Receiver operating characteristic (ROC) curves were used to evaluate the predictive capability of ALI and the Japanese CTO Registry (J-CTO) score for successful AR, with DeLong’ s test to compare model differences. Confusion matrix parameters were also used to assess model performance.

**Results:**

Patients in the successful AR group had significantly lower ALI values than those in the failed group (P = 0.012). Higher ALI was independently associated with lower odds of AR success in both unadjusted (OR = 0.624; 95% CI, 0.437–0.859; P = 0.006) and fully adjusted models (OR = 0.622; 95% CI, 0.416–0.879; P = 0.013). GAM confirmed an approximately linear inverse relationship between ALI and AR success (P = 0.013). Subgroup analyses showed consistent associations across clinical strata, with particularly strong effects in males and patients with prior PCI or MI. Sensitivity analysis revealed a significant dose–response trend across ALI quartiles (P for trend = 0.021), with the highest ALI quartile associated with significantly lower AR success (OR = 0.318; 95% CI, 0.121–0.96; P = 0.017). ROC curve analysis demonstrated that ALI alone had fair predictive performance for AR success (AUC = 0.608; 95% CI, 0.524–0.692), with high specificity (0.970) but low sensitivity (0.106; F1 = 0.182). The J-CTO score improved discrimination (AUC = 0.760; 95% CI, 0.696–0.823), and combining ALI with J-CTO further enhanced predictive ability (AUC = 0.798; 95% CI, 0.736–0.860; sensitivity = 0.439, specificity = 0.864, F1 = 0.513). DeLong’ s test confirmed a statistically significant AUC improvement for the combined model versus J-CTO alone (ΔAUC = 0.038; 95% CI, 0.007–0.069; *P* = 0.017), indicating additional prognostic value of ALI.

**Conclusions:**

ALI is independently and inversely associated with the success of AR in CTO lesions. As a composite biomarker of inflammation and nutrition, ALI may help identify patients with lower probability of AR success and, when combined with the J-CTO score, enhance preprocedural assessment and procedural planning.

## Introduction

 Chronic total occlusion (CTO) of the coronary artery is one of the most complex lesion types in coronary artery disease [1], with a detection rate of approximately 15%–20% among patients undergoing coronary angiography [2, 3]. Although CTO lesions do not always cause overt symptoms, their presence is closely associated with increased risks of adverse cardiovascular events and mortality [4, 5]. Percutaneous coronary intervention (PCI) for CTO remains one of the most technically demanding procedures in interventional cardiology.

Among available treatment strategies, the antegrade approach is generally preferred due to its direct path, relatively simple techniques, lower consumption of devices, and reduced risk of complications [6, 7]. However, in complex CTO cases, the antegrade route often fails due to unfavorable anatomical conditions, necessitating the use of the retrograde strategy, which carries a significantly higher complication risk. Studies have shown that the overall in-hospital incidence of major adverse cardiovascular events (MACE) for retrograde PCI is approximately 10.2%, markedly higher than the 4.7% seen with antegrade PCI, with all periprocedural deaths (0.8%) occurring in the retrograde group [8]. Retrograde procedures are also associated with a clinical coronary perforation rate of about 5.8% and an in-hospital MACE rate of approximately 3.5%, including severe complications such as cardiac tamponade and vessel perforation [9].

Coronary revascularization has been shown to improve clinical outcomes in patients with chronic coronary syndromes compared with optimal medical therapy. A recent systematic review and meta-analysis demonstrated that revascularization is associated with lower risks of cardiovascular mortality and myocardial infarction. However, the magnitude of benefit varies across patient subgroups, and evidence in complex lesions such as chronic total occlusion remains less consistent. These findings highlight the clinical importance of optimizing revascularization strategies in complex coronary artery disease [10]. In clinical practice, multiple switches between antegrade and retrograde strategies are often required during the revascularization of complex CTO lesions. This back-and-forth significantly prolongs the procedure time, often exceeding 2 h on average, and success rates decline sharply when guidewire manipulation exceeds 30 min [11]. Additionally, the incidence of related complications, including perforation, guidewire entrapment, and cardiac tamponade, also increases accordingly [12]. In this context, identifying factors that are associated with the success of antegrade recanalization (AR) holds substantial clinical value. Such identification not only facilitates preoperative patient evaluation and procedural planning but also assists operators in formulating more precise strategies, potentially reducing unnecessary retrograde attempts, lowering the risk of procedure-related complications, and ultimately enhancing patient outcomes.

The Advanced Lung Cancer Inflammation Index (ALI) is a composite index derived from body mass index (BMI), serum albumin level, and the neutrophil-to-lymphocyte ratio (NLR), and reflects both the nutritional status and systemic inflammatory response in patients [13]. Initially developed to assess survival in patients with advanced non-small cell lung cancer, ALI has more recently been applied in cardiovascular diseases, including predicting long-term prognosis and procedural risks in patients with coronary heart disease [13, 14]. However, the potential association between ALI and the success of AR in CTO lesions remains unexplored, and no systematic evaluations are currently available.

In this study, we hypothesize that ALI, as a composite indicator reflecting systemic inflammation and nutritional status, may be associated with successful antegrade recanalization. Given that ALI encompasses multiple physiological dimensions potentially influencing procedural outcomes—such as lesion complexity, endothelial function, and overall physiological resilience—its preprocedural assessment may offer additional value in CTO-PCI strategy formulation. Notably, in this study, successful AR is defined as the final recanalization of the CTO lesion via the antegrade approach, regardless of whether the retrograde route was attempted during the procedure and irrespective of the order of strategy application. This outcome-oriented definition emphasizes the ultimate route of success rather than the initial strategy, aligning more closely with real-world clinical practice and facilitating a more objective analysis of the relationship between preprocedural factors and antegrade success. By identifying the potential association between ALI levels and AR success, this study may aid in refining patient stratification, optimizing procedural strategies, improving procedural efficiency, minimizing operative risks, and ultimately enhancing patient prognosis.

## Materials and methods

### Study design and population

This retrospective cross-sectional study was conducted at the Department of Cardiology, Beijing Chaoyang Hospital, Capital Medical University. Consecutive patients diagnosed with CTO who underwent PCI between May 2024 and December 2025 were screened. A total of 252 patients were initially enrolled. After excluding those with incomplete angiographic data or missing parameters required for the calculation of the ALI, 198 patients were included in the final analysis.

In this study, AR success was defined as final successful revascularization of the occluded coronary artery via the antegrade approach, regardless of the number or sequence of previous antegrade or retrograde attempts. AR failure was defined as failure to achieve revascularization through the antegrade route, including both complete failure to open the occlusion and cases where the vessel was ultimately recanalized via a retrograde approach after unsuccessful antegrade attempts. Based on this definition, patients were categorized into the successful AR group (*n* = 132) and the failed AR group (*n* = 66) according to the final strategy that achieved revascularization.

**Fig. 1:**
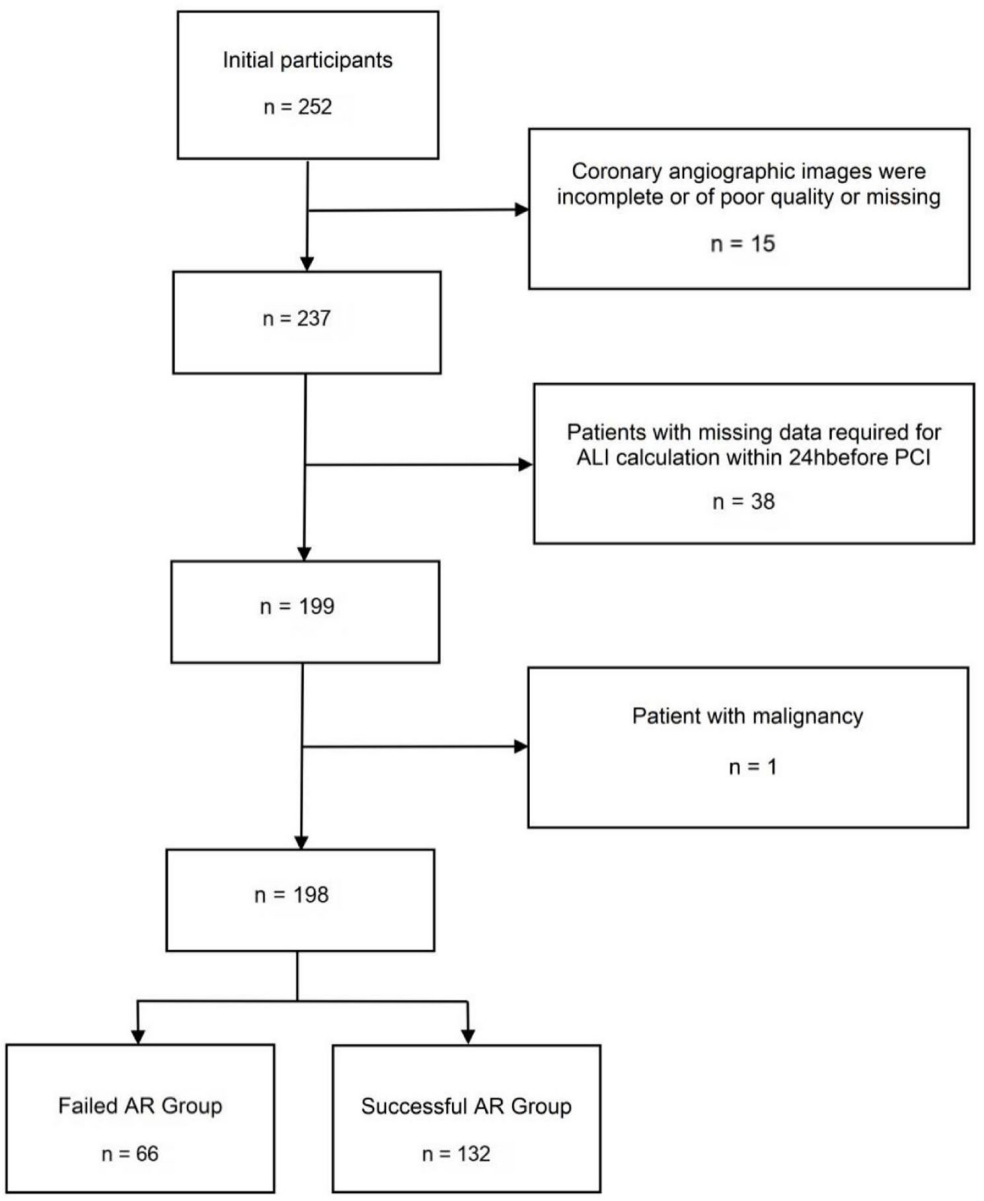
Detailed flowchart of patient selection

Inclusion criteria were: age ≥ 18 years, angiographically confirmed CTO, availability of ALI-related parameters, including BMI, ALB, NE, and LYM. And complete procedural records. Exclusion criteria included incomplete or poor-quality angiographic imaging, missing data for ALI calculation, and patients with severe dysfunction of major organs, autoimmune diseases, malignancies or acute infections or inflammatory conditions. A detailed flowchart of patient selection is presented in Fig. 1.

### Diagnostic criteria for coronary CTO

CTO of a coronary artery was defined as a complete blockage with no antegrade flow (TIMI grade 0) for an estimated duration of at least three months. The duration of occlusion was assessed based on clinical presentation, prior coronary angiography, or the first onset of symptoms suggestive of myocardial ischemia in the target vessel territory. In cases where the exact time of occlusion was unclear, a combination of patient history and ancillary findings was used for estimation.

Typical angiographic features of CTO included a blunt or tapered proximal cap, absence of antegrade contrast passage, presence of bridging collaterals, and retrograde filling of the distal vessel through collateral channels. All angiograms were independently reviewed by two experienced interventional cardiologists to confirm the diagnosis.

### Calculation of the Japanese CTO registry (J-CTO) score

The J-CTO score was derived from coronary angiographic data, with lesion characteristics independently evaluated by two experienced interventional cardiologists; any discrepancies were resolved through adjudication by a third expert. Following the original criteria, one point was assigned for each of five angiographic features: a blunt proximal cap (absence of a tapered stump), visible calcification within the occluded segment prior to contrast injection involving at least one vessel wall, marked vessel angulation exceeding 45° within the occlusion, an occlusion length of 20 mm or greater measured along the vessel’s centreline, and a history of prior unsuccessful PCI for the same lesion. The cumulative score ranged from 0 to 5.

### Measurement of clinical variables

Demographic variables including age, gender, height, and weight were recorded at admission. BMI was calculated based on height and weight measured at admission. Comorbidities such as hypertension, diabetes mellitus (DM), peripheral artery disease (PAD), Prior MI, Prior PCI, and CHD Family History were identified based on past medical records and patient-reported history. Smoking status was documented as current or prior smoker.

Routine blood tests were performed within 24 h before the procedure. Hematological parameters, including white blood cell count (WBC), hemoglobin (HGB), neutrophil count (NE), lymphocyte count (LYM), and platelet count (PLT), were measured using an automated hematology analyzer following standard operating procedures. Serum biochemical markers, including albumin (ALB), glucose (GLU), creatinine (CREA), urea (UREA), uric acid (UA), electrolytes such as sodium (Na), potassium (K), chloride (Cl), calcium (Ca), and phosphorus (P), liver enzymes such as alanine aminotransferase (ALT) and aspartate aminotransferase (AST), lipid profiles including total cholesterol (TC), triglycerides (TG), high-density lipoprotein (HDL), and low-density lipoprotein (LDL), and cardiac biomarkers including creatine kinase-MB (CK-MB), troponin I, and B-type natriuretic peptide (BNP), were also tested by the same department using a clinical chemistry analyzer according to institutional protocols.

Transthoracic echocardiography was performed before the intervention using a standard cardiac ultrasound system. Parameters assessed included left ventricular posterior wall thickness (LVPWD), interventricular septal thickness (IVSD), left ventricular internal diameter in systole (LVIDs) and diastole (LVIDd), E/A ratio (EA), and left ventricular ejection fraction (EF). All echocardiographic evaluations were performed and interpreted by experienced cardiologists in accordance with routine clinical protocols, and were blinded to patient outcomes.

After data cleaning, demographic information, comorbidities, and key variables required for ALI calculation were complete. Only a portion of laboratory test results had missing values, all with a missing rate of less than 15%. To minimize bias and retain sample size for subsequent analyses, data imputation was performed using a combination of multiple imputation and median imputation methods.

### ALI calculation method

The ALI was calculated using the following formula:

$$\mathrm{ALI}=\mathrm{BMI}\times\mathrm{A}\mathrm{LB}\times\left(\frac{\mathrm{LYM}}{\mathrm{N}\mathrm{E}}\right)$$

where BMI was calculated as weight in kilograms divided by height in meters squared (kg/m²); ALB represents serum albumin concentration (g/dL); LYM is the absolute lymphocyte count; and NE is the absolute neutrophil count, both expressed in ×10⁹/L. All laboratory values were obtained from blood samples collected within 24 h prior to the procedure.

### Statistical analysis

All statistical analyses were performed using R software (version 4.5.0). Normality of continuous variables was assessed using the Kolmogorov–Smirnov test. Normally distributed variables were expressed as mean ± standard deviation and compared using the independent samples t-test. Non-normally distributed variables were presented as Q2(Q1-Q3) and compared using the Mann–Whitney U test. Categorical variables were summarized as counts and percentages, and compared using the chi-square test.

Univariable and multivariable logistic regression analyses were conducted to assess the association between ALI and successful AR, with stepwise adjustment for potential confounders. Subgroup analyses were performed across stratified variables including age, gender, comorbidities, and medical history. A generalized additive model (GAM) was used to explore potential nonlinear associations between ALI and successful AR. Sensitivity analysis was conducted using ALI quartiles to evaluate dose–response relationships. The predictive capability of ALI and J-CTO Score for successful AR was evaluated using receiver operating characteristic (ROC) curves. The differences between the ROC models were compared using the DeLong test. The model’s performance was further evaluated using confusion matrix parameters, including accuracy, sensitivity, specificity, and F1 score. A two-sided p-value < 0.05 was considered statistically significant.

## Results

### Comparison of baseline characteristics between successful and failed AR groups

A total of 198 patients were included, with 132 in the successful AR group and 66 in the failed AR group. Patients in the successful group had significantly lower body weight and BMI compared with those in the failed group. Regarding laboratory findings, lymphocyte count and HDL levels were higher in the successful group. Echocardiographic measurements showed smaller LVID-d in patients with successful AR. The J-CTO score and prior PCI were also different between groups. Importantly, the ALI was significantly lower in the successful group (*P* = 0.015). As shown in Table 1.

**Table 1:** Baseline characteristics between Failed and Successful AR groups

Variables	Over all (*n* = 170)	Failed AR (*n* = 60)	Successful AR (*n* = 110)	*P*
Continuous variables, mean ± SD / Q2 (Q1-Q3)				
Age (years)	60.64 ± 9.54	60.20 ± 10.56	60.86 ± 9.02	0.661
Height (cm)	168.38 ± 8.20	169.05 ± 8.48	168.05 ± 8.07	0.432
Weight (kg)	74.64 ± 14.49	79.14 ± 12.82	72.39 ± 14.79	**0.001**
BMI (kg/m²)	26.24 ± 4.23	27.67 ± 4.09	25.52 ± 4.14	**< 0.001**
HR (bpm)	74.42 ± 10.30	74.03 ± 10.27	74.61 ± 10.35	0.708
SBP (mmHg)	128.89 ± 19.52	130.08 ± 18.36	128.30 ± 20.12	0.535
DBP (mmHg)	75.93 ± 10.65	75.79 ± 10.21	76.01 ± 10.89	0.889
WBC (×10⁹/L)	7.00 ± 2.21	7.44 ± 2.60	6.78 ± 1.96	0.074
HGB (g/L)	138.16 ± 15.37	138.89 ± 16.11	137.80 ± 15.04	0.645
NE (×10⁹/L)	4.19 (3.23, 5.49)	4.20 (3.60, 5.35)	4.19 (3.14, 5.50)	0.453
LYM (×10⁹/L)	1.54 (1.13, 2.01)	1.71 (1.29, 2.11)	1.46 (1.05, 1.88)	**0.007**
PLT (×10⁹/L)	204.97 ± 54.70	205.91 ± 52.95	204.51 ± 55.75	0.863
ALB (g/L)	42.10 (40.00, 44.20)	42.60 (40.02, 44.50)	41.50 (39.98, 44.10)	0.299
GLU (mmol/L)	5.95 (4.81, 7.68)	5.78 (4.62, 7.08)	6.12 (4.82, 7.95)	0.169
CREA (µmol/L)	74.00 (63.65, 86.15)	77.75 (64.22, 88.23)	73.10 (63.38, 84.12)	0.385
UREA (mmol/L)	6.90 (5.40, 8.32)	6.85 (5.38, 8.02)	6.90 (5.40, 8.42)	0.987
UA (µmol/L)	335.16 ± 93.63	336.24 ± 91.80	334.62 ± 94.87	0.908
TG (mmol/L)	1.59 (1.10, 2.20)	1.60 (1.12, 2.36)	1.59 (1.09, 2.09)	0.672
TC (mmol/L)	3.20 ± 0.87	3.06 ± 0.79	3.26 ± 0.91	0.112
HDL (mmol/L)	0.91 ± 0.23	0.86 ± 0.19	0.93 ± 0.24	**0.021**
LDL (mmol/L)	1.66 ± 0.72	1.63 ± 0.68	1.68 ± 0.74	0.644
Ca (mmol/L)	2.23 (2.16, 2.30)	2.23 (2.16, 2.28)	2.23 (2.16, 2.30)	0.283
P (mmol/L)	1.20 ± 0.19	1.22 ± 0.20	1.19 ± 0.19	0.332
Cl (mmol/L)	105.42 ± 2.75	105.54 ± 2.79	105.36 ± 2.73	0.652
K (mmol/L)	3.90 (3.70, 4.18)	4.00 (3.80, 4.20)	3.90 (3.70, 4.10)	0.274
Na (mmol/L)	140.40 (138.80, 141.50)	139.90 (138.72, 141.95)	140.50 (138.80, 141.40)	0.996
ALT (U/L)	28.00 (20.00, 38.00)	29.00 (22.25, 40.00)	27.00 (19.00, 37.00)	0.235
AST (U/L)	24.00 (20.00, 29.75)	23.00 (20.25, 31.75)	24.00 (20.00, 29.00)	0.662
LVPWD (mm)	10.00 (9.00, 11.00)	10.00 (9.00, 11.00)	10.00 (9.00, 10.62)	0.214
IVSD (mm)	10.00 (9.40, 11.15)	10.90 (10.00, 11.70)	10.00 (9.00, 11.00)	0.110
LVIDs (mm)	32.00 (29.00, 37.00)	33.50 (30.62, 39.00)	30.50 (29.00, 36.00)	**0.002**
LVIDd (mm)	49.00 (46.00, 53.00)	50.00 (47.00, 53.75)	48.00 (46.00, 53.00)	**0.036**
EF (%)	63.00 (51.00, 67.00)	60.50 (52.25, 64.75)	64.00 (51.00, 67.00)	0.084
CKMB (ng/mL)	1.83 (1.20, 2.95)	1.94 (1.29, 3.15)	1.74 (1.05, 2.82)	0.183
TNI (ng/mL)	0.04 (0.01, 9.95)	0.03 (0.01, 12.02)	0.04 (0.01, 9.65)	0.620
BNP (pg/mL)	76.54 (32.22, 216.48)	78.00 (30.43, 185.61)	76.06 (36.29, 223.42)	0.635
J-CTO score	2.00 (2.00, 3.00)	3.00 (3.00, 3.75)	2.00 (1.00, 3.00)	**< 0.001**
ALI_z	0.00 ± 1.00	0.30 ± 1.29	-0.15 ± 0.78	**0.012**
Categorical variables, n (%)				
Gender				
Female	165 (83.3%)	54 (81.8%)	111 (84.1%)	0.840
Male	33 (16.7%)	12 (18.2%)	21 (15.9%)	
Smoking				
No	78 (39.4%)	19 (28.8%)	59 (44.7%)	**0.045**
Yes	120 (60.6%)	47 (71.2%)	73 (55.3%)	
PAD				
No	111 (56.1%)	39 (59.1%)	72 (54.5%)	0.649
Yes	87 (43.9%)	27 (40.9%)	60 (45.5%)	
DM				
No	107 (54.0%)	30 (45.5%)	77 (58.3%)	0.118
Yes	91 (46.0%)	36 (54.5%)	55 (41.7%)	
Hypertension				
No	60 (30.3%)	21 (31.8%)	39 (29.5%)	0.870
Yes	138 (69.7%)	45 (68.2%)	93 (70.5%)	
CHD Family History				
No	162 (81.8%)	55 (83.3%)	107 (81.1%)	0.845
Yes	36 (18.2%)	11 (16.7%)	25 (18.9%)	
Prior PCI				
No	138 (69.7%)	42 (63.6%)	96 (72.7%)	0.251
Yes	60 (30.3%)	24 (36.4%)	36 (27.3%)	
Prior MI				
No	147 (74.2%)	49 (74.2%)	98 (74.2%)	1.000
Yes	51 (25.8%)	17 (25.8%)	34 (25.8%)	

*AR* Angiographic Recanalization, *BMI* Body Mass Index, *HR *Heart Rate, *SBP *Systolic Blood Pressure, *DBP *Diastolic Blood Pressure, *WBC *White Blood Cell Count, *HGB *Hemoglobin, *NE *Neutrophils, *LYM *Lymphocytes, *PLT *Platelet Count, *ALB* Albumin, *GLU *Glucose, *TBIL *Total Bilirubin, *DBIL *Direct Bilirubin, *ALT *Alanine Aminotransferase, *AST *Aspartate Aminotransferase, *BUN *Blood Urea Nitrogen, *Cr *Creatinine, *CK *Creatine Kinase, *CK-MB *Creatine Kinase-MB, *Myo *Myoglobin, *TnT *Troponin T, *BNP * B-type Natriuretic Peptide, *INR *International Normalized Ratio, *PT *Prothrombin Time, *APTT *Activated Partial Thromboplastin Time, *PCT *Procalcitonin, *CRP* C-reactive Protein, *LVEF *Left Ventricular Ejection Fraction, *RWMA *Regional Wall Motion Abnormality, *CHD *Coronary Heart Disease, *MI *Myocardial Infarction, *PCI *Percutaneous Coronary Intervention, *PAD *Peripheral Artery Disease, *DM*  Diabetes Mellitus

### Association between ALI and successful AR in multivariable logistic regression analysis

**Fig. 2:**
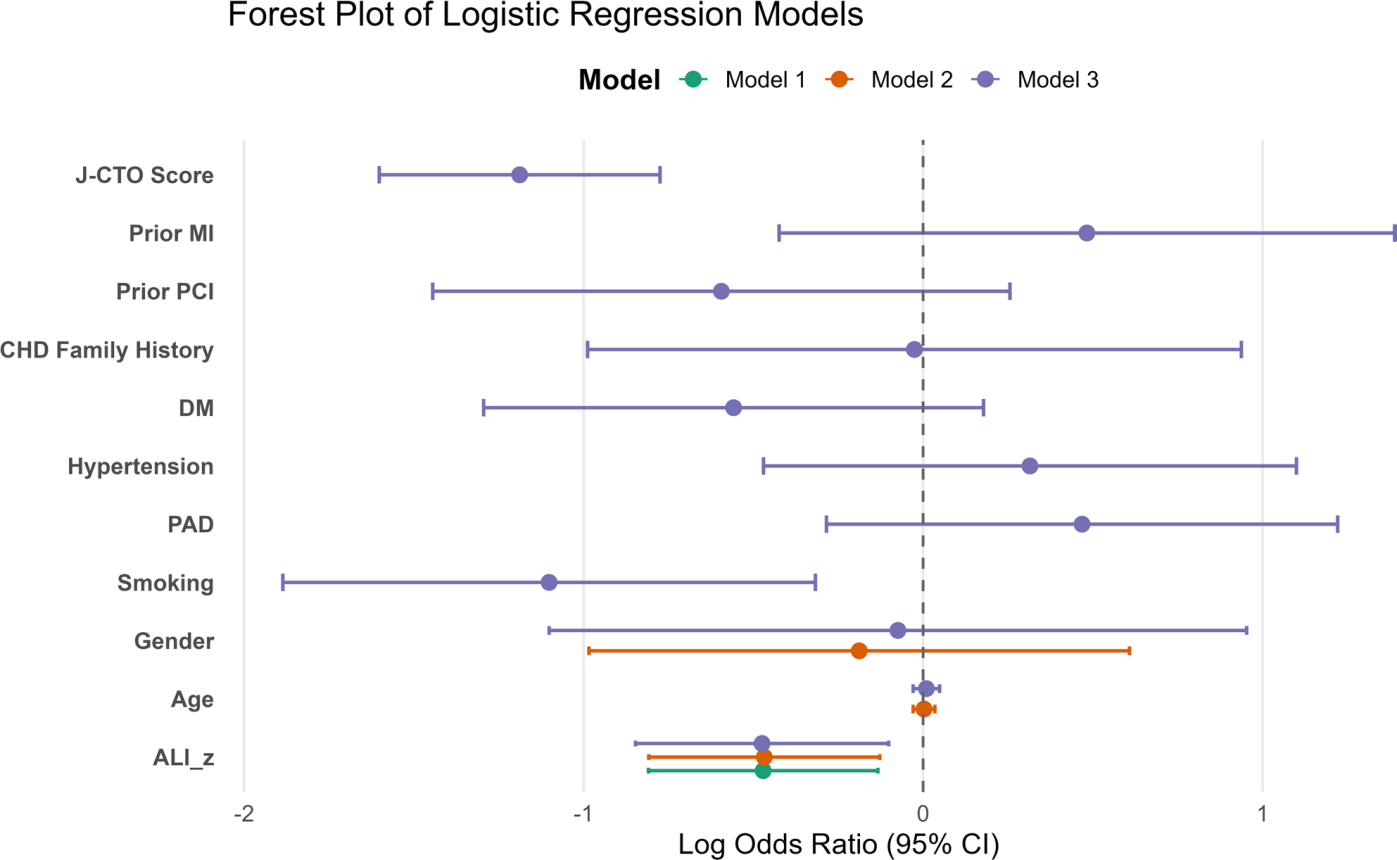
Forest Plot for 3 Logistic Regression of the Association Between ALI and Successful AR

In unadjusted logistic regression analysis, ALI was significantly associated with successful AR (OR = 0.624; 95% CI, 0.437–0.859; *P* = 0.006). This association remained significant after adjusting for age and gender (OR = 0.626; 95% CI, 0.437–0.862; *P* = 0.007). In the fully adjusted model including age, sex, smoking, PAD, hypertension, DM, CHD family history, prior PCI, prior MI and J-CTO Score. ALI remained significantly associated with successful AR (OR = 0.622; 95% CI, 0.416–0.879; *P* = 0.013). Smoking was also significantly associated with AR success (OR = 0.332; 95% CI, 0.147–0.712; *P* = 0.006), suggesting a negative impact. In addition, a higher J-CTO score was strongly associated with lower odds of successful AR (OR = 0.305; 95% CI, 0.196–0.450; *P* < 0.001).As shown in Table 2; Fig. 2.

**Table 2:** Association Between ALI and AR success in Multivariable Logistic Regression Models

Variable	OR(95% CI)	Std.error	Z	*P*
Model 1 (unadjusted)				
ALI_z	0.624 (0.437–0.859)	0.172	-2.733	**0.006**
Model 2 (adjusted for age and gender)				
ALI_z	0.626 (0.437–0.862)	0.174	-2.695	**0.007**
Age	1.003 (0.971–1.035)	0.016	0.166	0.869
Gender	0.828 (0.378–1.880)	0.406	-0.463	0.643
Model 3 (fully adjusted)				
ALI_z	0.622 (0.416–0.879)	0.190	-2.493	**0.013**
Age	1.010 (0.971–1.050)	0.020	0.482	0.630
Gender	0.928 (0.335–2.664)	0.524	-0.142	0.887
Smoking	0.332 (0.147–0.712)	0.400	-2.753	**0.006**
PAD	1.597 (0.759–3.444)	0.384	1.219	0.223
Hypertension	1.370 (0.622–3.014)	0.401	0.786	0.432
DM	0.572 (0.271–1.189)	0.376	-1.486	0.137
CHD Family History	0.975 (0.377–2.636)	0.491	-0.052	0.959
Prior PCI	0.552 (0.233–1.289)	0.434	-1.370	0.171
Prior MI	1.620 (0.665–4.123)	0.463	1.043	0.297
J-CTO Score	0.305 (0.196–0.450)	0.211	-5.632	**< 0.001**

*CHD* Coronary Heart Disease, *MI *Myocardial Infarction, *PCI *Percutaneous Coronary Intervention, *PAD *Peripheral Artery Disease, *DM*  Diabetes Mellitus

### Subgroup analyses of the association between ALI and successful AR

**Fig. 3:**
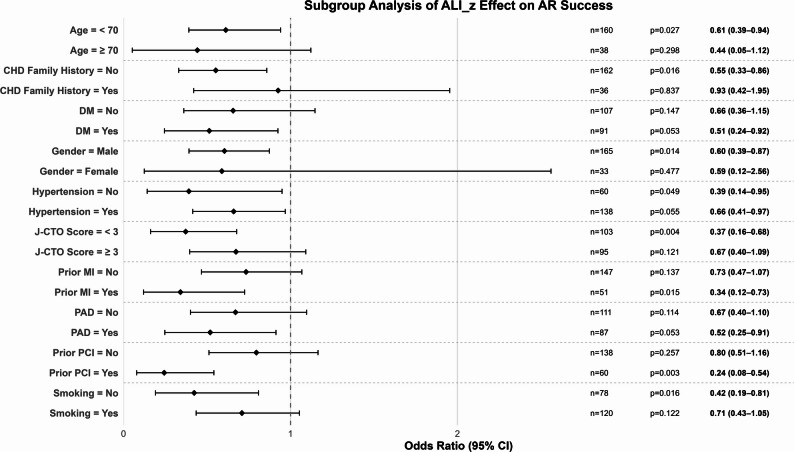
Forest Plot for Subgroup Analyses of the Association Between ALI and Successful AR

Subgroup analyses stratified by age, gender, smoking status, PAD, hypertension, DM, CHD family history, prior PCI, prior MI and J-CTO Score demonstrated generally consistent associations between ALI and successful AR across most subgroups. Notably, significant associations were observed in males (OR = 0.604, 95% CI 0.392–0.873, *p* = 0.014), non-smokers (OR = 0.423, 95% CI 0.191–0.808, *p* = 0.016), patients without hypertension (OR = 0.391, 95% CI 0.141–0.949, *p* = 0.049), and those with prior PCI (OR = 0.243, 95% CI 0.079–0.541, *p* = 0.003) or prior MI (OR = 0.341, 95% CI 0.120–0.725, *p* = 0.003). A significant association was also observed among patients with a J-CTO score < 3 (OR = 0.372, 95% CI 0.162–0.678, *p* = 0.004). While effect sizes varied across subgroups, the overall pattern supports ALI as a potentially robust biomarker associated with AR success. As shown in Fig. 3; Table 3.

**Table 3:** Subgroup analysis of ALI on AR success

Variable	Level	*n*	OR(95% CI)	*P*
Age	< 70	160	0.612 (0.391–0.940)	**0.027**
	≥ 70	38	0.442 (0.052–1.122)	0.298
Gender	Male	165	0.604 (0.392–0.873)	**0.014**
	Female	33	0.589 (0.124–2.560)	0.477
Smoking	No	78	0.423 (0.191–0.808)	**0.016**
	Yes	120	0.709 (0.435–1.053)	0.122
PAD	No	111	0.670 (0.401–1.096)	0.114
	Yes	87	0.519 (0.247–0.913)	0.053
Hypertension	No	60	0.391 (0.142–0.949)	**0.049**
	Yes	138	0.659 (0.414–0.969)	0.055
DM	No	107	0.656 (0.361–1.147)	0.147
	Yes	91	0.513 (0.245–0.925)	0.053
CHD Family History	No	162	0.552 (0.330–0.858)	**0.016**
	Yes	36	0.926 (0.420–1.954)	0.837
Prior PCI	No	138	0.795 (0.512–1.165)	0.257
	Yes	60	0.243 (0.079–0.541)	**0.003**
Prior MI	No	147	0.734 (0.467–1.068)	0.137
	Yes	51	0.341 (0.120–0.725)	**0.015**
J-CTO Score	< 3	103	0.372 (0.162–0.678)	**0.004**
	≥ 3	95	0.673 (0.396–1.092)	0.121

*CHD *Coronary Heart Disease, *MI *Myocardial Infarction, *PCI *Percutaneous Coronary Intervention, *PAD *Peripheral Artery Disease, *DM* Diabetes Mellitus

### GAM analysis of the association between ALI and successful AR

**Fig. 4:**
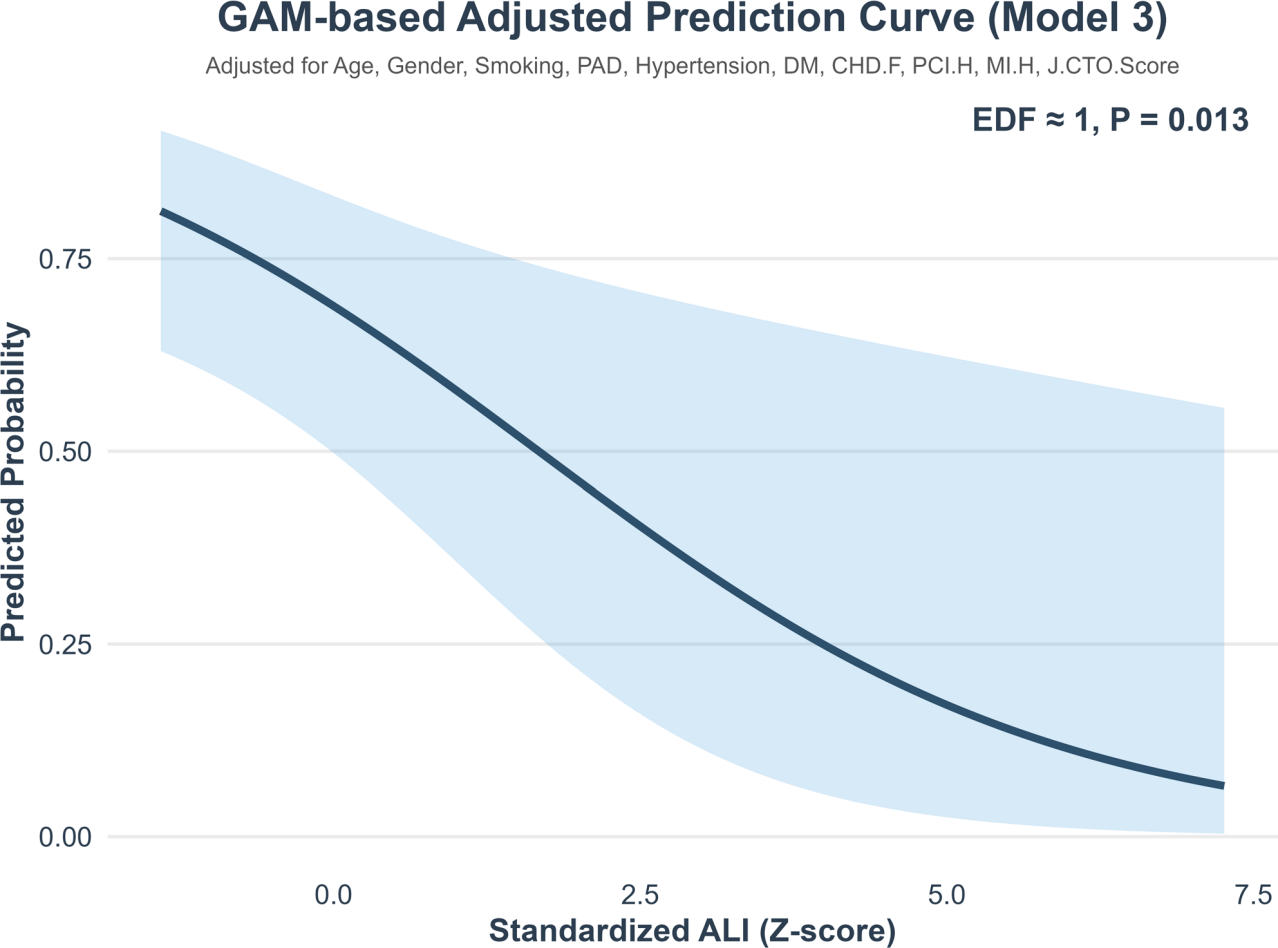
GAM Curve of the Association Between ALI and Successful AR

To explore the potential nonlinear relationship between ALI and successful antegrade recanalization, a GAM was fitted with full covariate adjustment. The smooth term for ALI yielded an effective degrees of freedom (EDF) of 1.000, indicating an approximately linear association. This relationship was statistically significant (*p* = 0.013), suggesting a consistent inverse association between ALI levels and the likelihood of achieving AR. As shown in Fig. 4.

### Sensitivity and dose–response trend analyses of ALI and successful AR

**Fig. 5:**
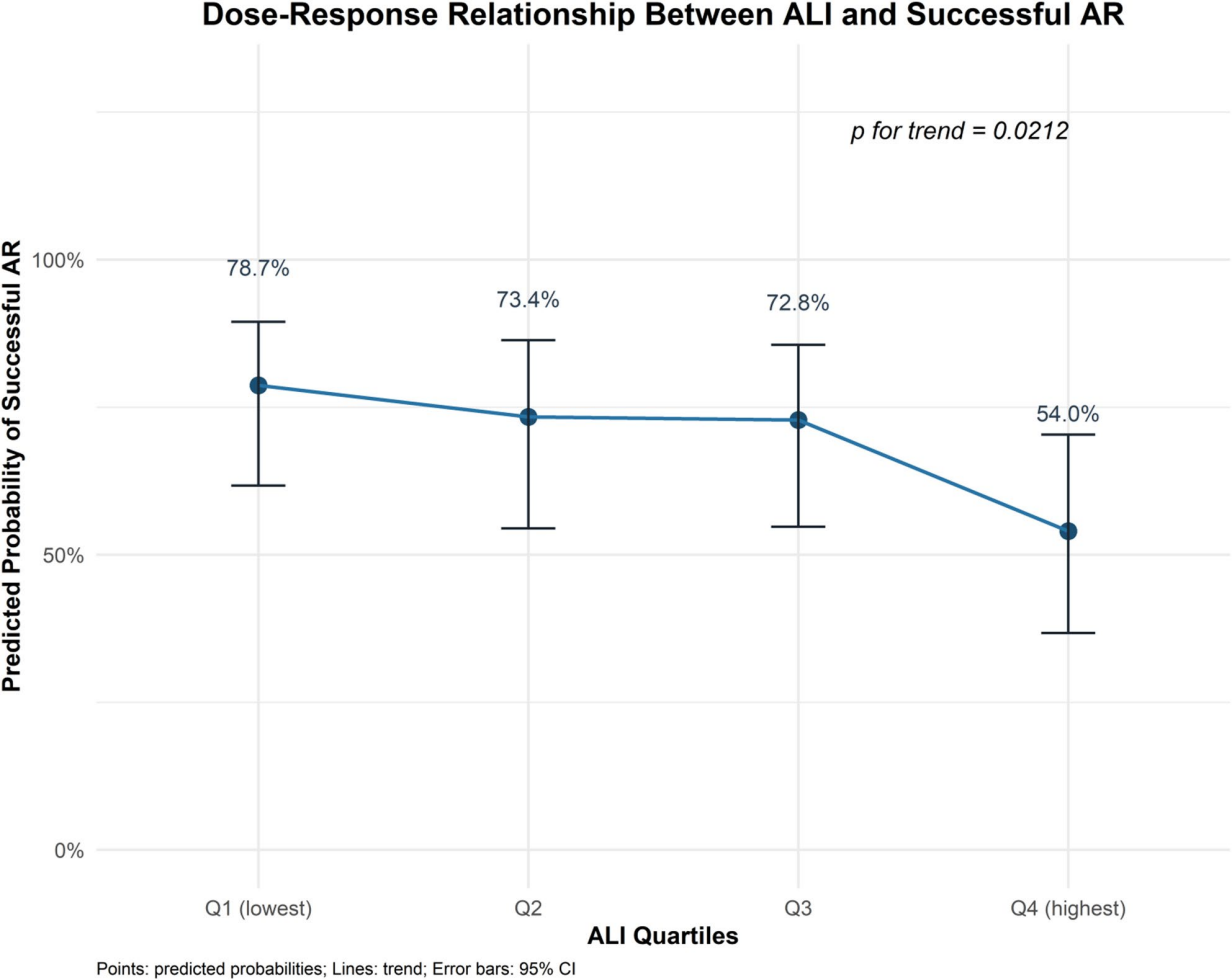
Dose-response relationship between ALI and successful AR

In the sensitivity analysis using ALI quartiles, the association between ALI and AR remained consistent. Compared with the lowest quartile (Q1), the OR for successful AR were 0.744 (95% CI: 0.286–1.890, *p* = 0.538) for Q2 and 0.725 (95% CI: 0.277–1.860, *p* = 0.504) for Q3, both of which were not statistically significant. However, patients in the highest quartile (Q4) had a significantly lower likelihood of successful AR, with an OR of 0.318 (95% CI: 0.121–0.796, *p* = 0.017). A significant linear trend was observed across ALI quartiles (p for trend = 0.021), indicating a potential dose–response relationship between decreasing ALI levels and increased probability of successful AR. As shown in Table 4; Fig. 5.

**Table 4:** Sensitivity Analysis and Trend Test

Variable	OR(95% CI)	Std.error	t	*P*
ALI Quartile				
Q1	Reference	Reference	Reference	Reference
Q2	0.744 (0.286–1.890)	0.478	-0.618	0.538
Q3	0.725 (0.277–1.860)	0.482	-0.669	0.504
Q4	0.318 (0.121–0.796)	0.478	-2.400	**0.017**
Trend Test	0.703 (0.517–0.944)	0.153	-2.310	**0.021**

### Prediction of successful AR using ALI, J-CTO, and Combined Models

**Fig. 6:**
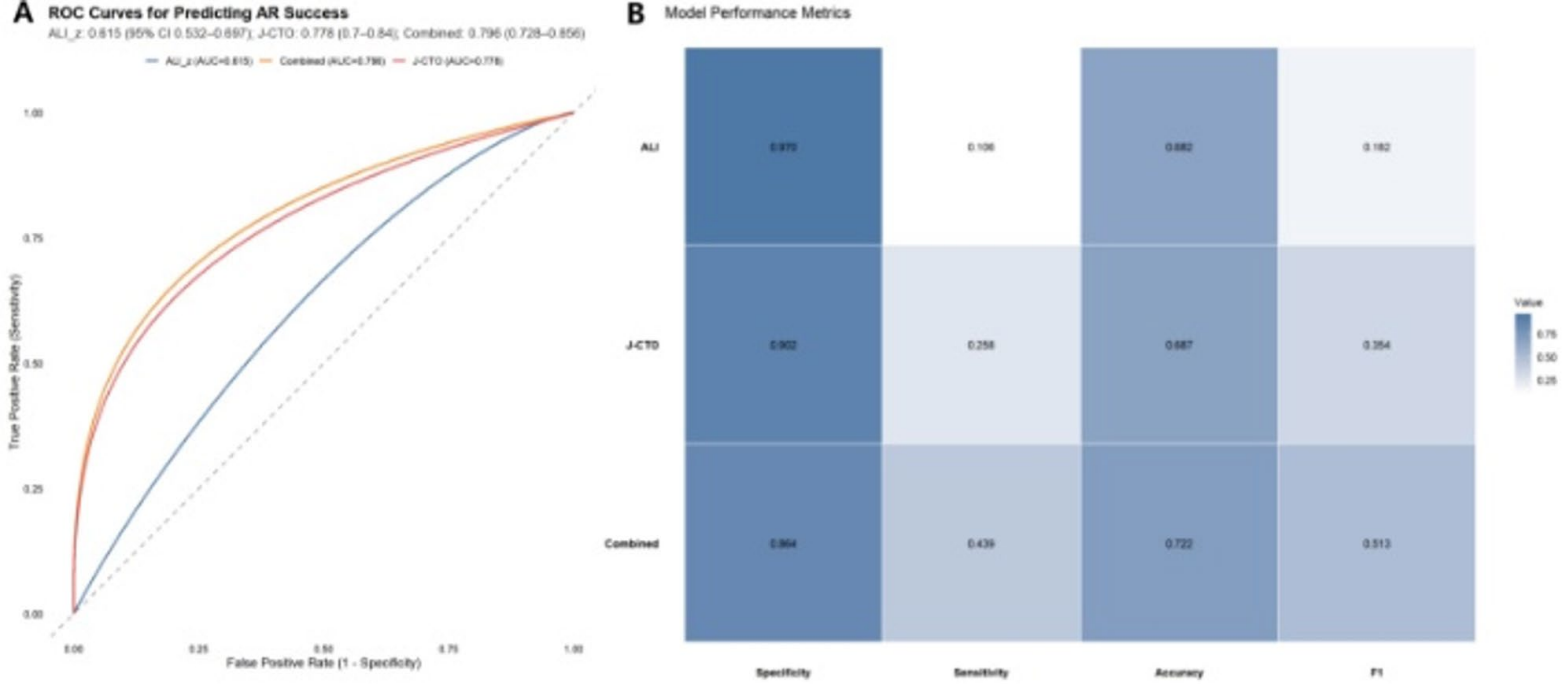
Performance evaluation of successful ar prediction models (**A** ROC Curve Comparison of ALI, J-CTO, and Combined Model; **B** Confusion Matrix Heatmap for Successful AR Prediction)

ROC curve analysis indicated that the ALI alone demonstrated fair predictive performance for AR success, with an AUC of 0.608 (95% CI: 0.524–0.692), high specificity (0.970), but low sensitivity (0.106), and an F1 score of 0.182. The J-CTO score showed improved discrimination, achieving an AUC of 0.760 (95% CI: 0.696–0.823), with a sensitivity of 0.258, specificity of 0.902, and an F1 score of 0.354. Combining ALI with the J-CTO score further enhanced predictive performance, yielding an AUC of 0.798 (95% CI: 0.736–0.860), sensitivity of 0.439, specificity of 0.864 accuracy of 0.722, and an F1 score of 0.513. DeLong’s test demonstrated a statistically significant improvement in AUC for the combined model compared with the J-CTO score alone (ΔAUC = 0.038; 95% CI: 0.007–0.069; *p* = 0.017), indicating that inclusion of ALI may provide additional prognostic value for predicting AR success. As shown in Fig. 6.

## Discussion

This retrospective cross-sectional study is the first to systematically investigate the association between the ALI and the success rate of AR in patients with coronary CTO. We observed that patients in the successful AR group had significantly lower body weight and BMI. Higher BMI is often associated with metabolic syndrome, insulin resistance, and chronic low-grade inflammation, characterized by elevated levels of cytokines such as TNF-α and IL-6 [15]. These inflammatory processes can drive vascular wall remodeling and fibrotic changes, increasing the rigidity of the occluded segment and making antegrade wiring more challenging [16]. Within the ALI components, although serum albumin levels did not differ significantly between groups, neutrophil and lymphocyte counts were both lower in the AR success group, suggesting a more balanced immune response and reduced inflammatory activation. Excessive inflammation is closely linked to plaque calcification [17–19], which is a well-established predictor of AR failure [20]. Prior studies have shown that severe calcification is associated with markedly lower antegrade crossing success rates [21]. Taken together, elevated ALI values may reflect enhanced collagen deposition and vascular calcification, contributing to increased resistance to guidewire passage. Conversely, low ALI may correspond to a lesion morphology that is more penetrable. However, given the retrospective cross-sectional design of this study, the observed association between ALI and AR success should not be interpreted as a direct causal relationship. It remains possible that lower ALI does not actively facilitate antegrade recanalization but instead reflects a more favorable systemic and vascular condition in patients with less complex CTO disease. In this context, ALI may serve primarily as a surrogate marker of the overall inflammatory and metabolic milieu rather than a direct determinant of procedural difficulty.

This observation suggests that ALI may reflect the underlying anatomical and biological characteristics of the occluded segment from multiple dimensions, including inflammation, nutrition, and metabolic state. Particularly in lesions with minimal tissue remodeling and preserved “softer” structures, a lower ALI may indicate more favorable conditions for antegrade crossing. Our subgroup analyses highlight the importance of ALI in predicting successful AR, particularly in patients with a J-CTO score < 3. In these individuals, who tend to have less complex CTO lesions, ALI likely reflects better endothelial function and vascular health, key factors for successful AR. The association between ALI and AR success was also notable in several other subgroups, including males, non-smokers, and those with a history of prior PCI, suggesting that ALI may offer valuable insights into patient prognosis across diverse clinical contexts. In contrast, the relationship between ALI and AR success was less consistent in patients with a J-CTO score ≥ 3, reflecting the dominant role of severe anatomical complexity. For these patients, factors such as calcification, lesion length, and tortuosity may override the functional effects captured by ALI. Nevertheless, ALI might still provide supplementary information regarding vascular health, potentially influencing procedural outcomes via endothelial function and microvascular regulation.These findings reinforce ALI as a complementary marker to the J-CTO score, particularly in less complex cases, and support its potential role in refining patient stratification for CTO revascularization.

The ROC analysis showed that ALI alone demonstrated fair discriminative ability for predicting AR success, with an AUC of 0.608. This finding is consistent with prior evidence [22] suggesting that systemic inflammatory biomarkers, although biologically informative, often lack sufficient specificity to reflect the anatomical and procedural complexity inherent to CTO recanalization. In contrast, the J-CTO score demonstrated superior predictive accuracy, with an AUC of 0.760, indicating better discrimination and clinical utility. This score, based on well-established angiographic parameters such as lesion calcification, tortuosity, and occlusion length, has been widely validated in the context of CTO treatment. Its relatively high specificity further reinforces its value as a decision-support tool in procedural planning [23, 24]. However, its sensitivity remained relatively limited, suggesting that anatomical scoring alone may not fully capture the multifactorial determinants of procedural success. To enhance predictive performance, we constructed a combined model integrating ALI with the J-CTO score. This composite model achieved a higher AUC of 0.798, together with a noticeable increase in sensitivity while maintaining acceptable specificity. Statistical comparison using DeLong’s test demonstrated that the combined model significantly outperformed the J-CTO score alone. These findings suggest that incorporating ALI into the conventional anatomical scoring system may provide additional prognostic information and improve the overall predictive performance for AR success.

Importantly, the modest ROC performance of ALI does not preclude its clinical relevance. In multivariable logistic regression analysis, ALI remained independently associated with successful AR after adjustment for demographic and clinical covariates, including the J-CTO score. This apparent discrepancy highlights the complementary nature of different statistical approaches. ROC analysis evaluates the overall discriminative ability of a predictor, whereas regression models identify independent associations after controlling for potential confounders. The relatively low sensitivity observed in the ROC analysis may reflect the nonspecific nature of systemic inflammation captured by ALI, which does not directly correspond to lesion-specific characteristics that determine procedural success. Nevertheless, its independent association with AR success suggests that ALI may reflect broader physiological conditions, such as endothelial function, inflammatory status, and microvascular reactivity, which may indirectly influence procedural tolerance and technical success.

Compared to previous studies on CTO-PCI outcomes, this is the first study to introduce ALI as a preprocedural risk assessment biomarker, adding novel and clinically relevant value. Prior research has predominantly focused on anatomical imaging characteristics—such as the J-CTO score [25], lesion length [26], degree of calcification [27], duration of occlusion [24], and procedural techniques [28]—to predict antegrade success. More recently, several studies [29–31] have employed machine learning models for prediction, though these too have largely relied on imaging parameters. Overall, existing literature has prioritized local lesion morphology, with little attention to systemic factors like inflammation or nutritional status. However, it is precisely these systemic conditions that shape the lesion’s biological behavior over time. Building on this rationale, our study introduces ALI into the AR prediction model and demonstrates its potential to enhance preprocedural risk stratification. By incorporating ALI into clinical decision-making frameworks, we may improve patient selection, optimize procedural planning, and ultimately increase procedural success rates. In addition, non-invasive imaging modalities have further expanded the preprocedural assessment of CTO lesions. Recent evidence [32] highlights the important role of advanced imaging techniques in providing detailed characterization of plaque composition and lesion complexity, thereby improving risk stratification and procedural planning. In this context, future research should aim to integrate non-invasive imaging modalities with invasive angiographic assessment and systemic biomarkers for a more comprehensive evaluation of both patient status and lesion behavior. Incorporating such multidimensional data into clinical decision-making frameworks may improve patient selection, enhance procedural planning, and ultimately increase the likelihood of procedural success.

Nonetheless, several limitations of this study shoud be acknowledged. First, this was a retrospective, single-center study, which limits causal inference and may introduce selection bias. Although we performed comprehensive multivariable adjustments, residual confounding cannot be fully excluded. Second, the sample size was relatively modest, which may affect the statistical power and robustness of our results. Third, Only the J-CTO score was included as the imaging parameter, and the lack of detailed morphological or intravascular imaging data on CTO lesions precluded analysis of the interplay between ALI and specific anatomical features, limiting mechanistic insight. Fourth, operator skill and experience, which are known to influence CTO-PCI success rates, were not accounted for in this study. Differences in individual operator proficiency or annual case volume may have introduced additional variability in procedural outcomes.

## Conclusion

In conclusion, this study is the first to reveal a significant and consistent association between ALI and the success of AR in CTO lesions. As a composite index reflecting systemic inflammation and nutritional status, ALI may serve as a valuable adjunctive marker for preprocedural assessment and procedural planning. Although ALI alone demonstrated fair discriminative ability for predicting AR success in ROC analysis, its integration with the J-CTO score significantly improved predictive performance. Incorporating ALI into CTO risk stratification models may facilitate more personalized interventional strategies, optimize procedural planning, and improve procedural outcomes.

## Data Availability

The datasets used and/or analyzed during the current study are available from the corresponding author on reasonable request.
